# Automated Atrial Fibrillation Diagnosis by Echocardiography without ECG: Accuracy and Applications of a New Deep Learning Approach

**DOI:** 10.3390/diseases12020035

**Published:** 2024-02-09

**Authors:** Nelson Lu, Hooman Vaseli, Mobina Mahdavi, Fatemah Taheri Dezaki, Christina Luong, Darwin Yeung, Ken Gin, Michael Tsang, Parvathy Nair, John Jue, Marion Barnes, Delaram Behnami, Purang Abolmaesumi, Teresa S. M. Tsang

**Affiliations:** 1 Division of Cardiology, Department of Medicine, University of British Columbia, Vancouver, BC V5Z 1M9, Canada; nelsonlu@student.ubc.ca (N.L.);; 2 Department of Electrical and Computer Engineering, University of British Columbia, Vancouver, BC V5Z 1M9, Canada

**Keywords:** artificial intelligence, deep learning, atrial fibrillation, echocardiography

## Abstract

Background: Automated rhythm detection on echocardiography through artificial intelligence (AI) has yet to be fully realized. We propose an AI model trained to identify atrial fibrillation (AF) using apical 4-chamber (AP4) cines without requiring electrocardiogram (ECG) data. Methods: Transthoracic echocardiography studies of consecutive patients ≥ 18 years old at our tertiary care centre were retrospectively reviewed for AF and sinus rhythm. The study was first interpreted by level III-trained echocardiography cardiologists as the gold standard for rhythm diagnosis based on ECG rhythm strip and imaging assessment, which was also verified with a 12-lead ECG around the time of the study. AP4 cines with three cardiac cycles were then extracted from these studies with the rhythm strip and Doppler information removed and introduced to the deep learning model ResNet(2+1)D with an 80:10:10 training–validation–test split ratio. Results: 634 patient studies (1205 cines) were included. After training, the AI model achieved high accuracy on validation for detection of both AF and sinus rhythm (mean F1-score = 0.92; AUROC = 0.95). Performance was consistent on the test dataset (mean F1-score = 0.94, AUROC = 0.98) when using the cardiologist’s assessment of the ECG rhythm strip as the gold standard, who had access to the full study and external ECG data, while the AI model did not. Conclusions: AF detection by AI on echocardiography without ECG appears accurate when compared to an echocardiography cardiologist’s assessment of the ECG rhythm strip as the gold standard. This has potential clinical implications in point-of-care ultrasound and stroke risk stratification.

## 1. Introduction

Atrial fibrillation (AF) is the predominant arrhythmia encountered in clinical practice with growing prevalence worldwide due to the aging population [1]. Widely recognized for its increased risk of stroke, AF is also associated with significant cardiovascular morbidity and mortality [2]. Early and accurate diagnosis of this arrhythmia is essential for timely management. Contemporary guidelines agree on rhythm-based detection through a standard 12-lead electrocardiogram (ECG) or single-lead ECG tracing ≥ 30 s as the gold standard for diagnosis of AF [3,4,5]. Echocardiography, whether using cart-based ultrasound machines or point-of-care ultrasound (POCUS) devices, is an integral part of the initial evaluation of a patient with palpitations with or without documented AF/flutter and is highly valuable for screening for substrates of arrhythmia [6]. These investigations also provide excellent opportunities for detection of AF/flutter, as well as imaging substrates that are known to promote the development of such arrhythmias.

Over the years, the efficiency of AF diagnosis has been accelerated by technological advances in medicine, including the introduction of artificial intelligence (AI). ECGs have long since been subjected to early computer-aided interpretation algorithms for more than 50 years, but are now capable of achieving a high diagnostic performance comparable to that of trained cardiologists in classifying a broad range of distinct arrhythmias through the implementation of machine learning and use of deep learning neural networks [7]. The versatility of AI has also notably extended to the realm of echocardiography. This includes improved image acquisition and optimization, automated view classification, measurements, and finally detection and interpretation of disease [8,9]. AI development in these areas promises to increase efficiency and decrease operator variability between sonographers to improve the overall workflow for echocardiography clinicians. Deep learning algorithms for assessment of left ventricular dysfunction, valvular disease, and cardiomyopathy have been described in the literature [10]. Despite all these advances, automated rhythm detection on echocardiography has yet to be fully realized. To our knowledge, there is only one prior study from our centre featuring Echo-Rhythm Net, a semi-supervised deep learning-based framework on echocardiography with a diagnostic accuracy of 73–79% for AF based on training and testing with parasternal long-axis (PLAX) cines [11]. This technology can be of clinical relevance in point-of-care ultrasound (POCUS) where rhythm strips are unavailable, which may be used in settings where ECGs are not immediately accessible.

In this paper, we propose a deep learning AI model trained to identify AF with high diagnostic accuracy using apical-4-chamber (AP4) cines on echocardiography without the need for conventional ECG information.

## 2. Materials and Methods

### 2.1. Study Overview

Transthoracic echocardiography studies of consecutive patients ≥ 18 years old at our Canadian tertiary care centre were retrospectively reviewed for cardiac rhythms identified as AF or sinus rhythm, which were the dominant rhythms in our database (>95%). Analyses of other types of rhythms and patients with congenital heart disease or cardiac devices such as pacemakers were excluded. This study was approved by the institutional review board of the University of British Columbia (H20-00602) and complied with the guidelines set forth in the Declaration of Helsinki. A waiver of consent was obtained for collection and use of patient data for this retrospective review. The authors had full access to the data and have all read and agreed with the contents of the manuscript as written.

### 2.2. Echocardiography and ECG Inclusion Criteria

Two-dimensional (2D) echocardiographic imaging consisting of AP4 cine loops with exactly three cardiac cycles were included. At our centre, these studies were obtained by certified sonographers and read by level III-trained echocardiography cardiologists on the same day. The echo cine data were captured using a variety of ultrasound machines from different manufacturers and models, including GE (Vivid i, Vivid E9, Vivid7, and Vivid E95; Milwaukee, WI, USA) and Philips (iE33, SONOS, EPIQ 7C; Bothell, WA, USA), with the majority of the data obtained from the Philips iE33 model. Syngo Dynamics (Siemens Medical Solutions, Ann Arbor, MI, USA) was the archiving and analysis platform for this study, and TomTec was used for strain analyses. The gold standard for cardiac rhythm was the echocardiography cardiologists’ interpretation of the ECG rhythm strip at the time of echocardiogram, in addition to imaging assessment of the full study including Doppler. The rhythm was additionally confirmed with an external 12-lead ECG, reviewed by a staff cardiologist, around the time of each echocardiography study to account for similar irregular rhythms that may be undifferentiable on the echocardiogram rhythm strip alone. When possible, two consecutive ECGs around the time of echocardiography were examined to acknowledge the possibility of paroxysmal AF.

### 2.3. AI model Framework, Training Process, and Evaluation

**Figure 1:**
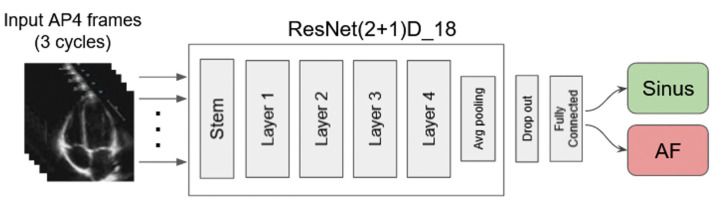
Deep learning model schematic diagram.

For the base deep learning model, we utilized ResNet(2+1)D, an open-source video-assessment convolutional residual neural network consisting of 18 deep layers. This model efficiently learns representations of video data by decomposing 3D convolution into pairs of 2D and 1D convolutions to extract spatial and temporal features, respectively, which minimizes overfitting [12] (Figure 1). The final output layer of the network was modified for two-class classification to identify AF or sinus rhythm as a binary output. Overall, this model contained around 31 million trainable parameters. The training procedure was carried out for 100 epochs using the Adam optimizer and weighted cross-entropy loss, assigning higher weights to less frequent labels to penalize classification errors more heavily. This approach mitigated the effects of imbalanced AF-sinus distribution in the dataset. The learning rate (LR) was initialized to 10^−3^, but it was halved whenever the performance on the validation set did not improve for 10 epochs. This process continued until the LR reached a minimum of 10^−5^.

The 2D AP4 cines for each patient study were extracted from Syngo Dynamics (VA40)/TomTec (Ultrasound Workspace) software and subsequently processed to remove the rhythm strip and additional Doppler information. These cines were introduced to the AI model in an 80:10:10 training–validation–test split ratio with mutually exclusive patients for deep learning. A total of 80% of studies were used for training of the AI model in batches of 8 cine clips. Each clip comprised three cardiac cycles extracted from a randomly selected echo cine and resized to 24 frames, each 112 by 112 pixels. Additionally, to prevent overfitting, each training batch was augmented by random rotation (−20° to 20°) and translation (up to 10% in horizontal and vertical directions). A total of 10% of studies were used as the validation set to identify the optimal configurations for the model and training procedure. The remaining 10% of studies were used for testing the best configured AI model to evaluate its generalizability to unseen data and its accuracy in identifying AF from sinus rhythm.

### 2.4. Statistical Analysis

Continuous variables of baseline characteristics are reported as the mean ± one standard deviation and were compared using two-tailed Student’s *t*-tests. The predictions made by the AI model when applied to the 2D AP4 cines in terms of identifying AF or sinus rhythm were compared to the original diagnosis by the echocardiography cardiologist in the dataset. Precision, recall, F1-scores, and the area under the receiver operating characteristic curve (AUROC) were calculated to assess the performance of the AI model in accurately diagnosing the rhythm, which were then used to determine the best model configuration and training hyper-parameters. The F1-score is the harmonic mean of precision and recall and calculated with the following formula: F1-score = 2 × (Precision × Recall)/(Precision + Recall). In comparison to calculating for accuracy, the F1-score provides a better assessment of the model’s performance when accounting for false positives and false negatives in imbalanced datasets.

### 2.5. Qualitative Analysis

We conducted a qualitative analysis of the AI model’s ability to perceive and classify the rhythm of the input echo cine as either AF or sinus. To assess the model’s sensitivity to spatio-temporal features, we removed a window of size 10 by 10 pixels across 5 consecutive frames and measured the change in rhythm prediction. We repeated this occlusion-based importance estimation for all the spatio-temporal features by sliding this window across all regions and frames in the given echo cine. Furthermore, we estimated the frame-level temporal importance by expanding the height and width of the aforementioned window to match the height and width of the echo cine. This information was then transformed into heatmaps, which were superimposed on the echo cine and evaluated for their clinical relevance in rhythm diagnosis.

## 3. Results

### 3.1. AI Training and Validation

**Figure 2:**
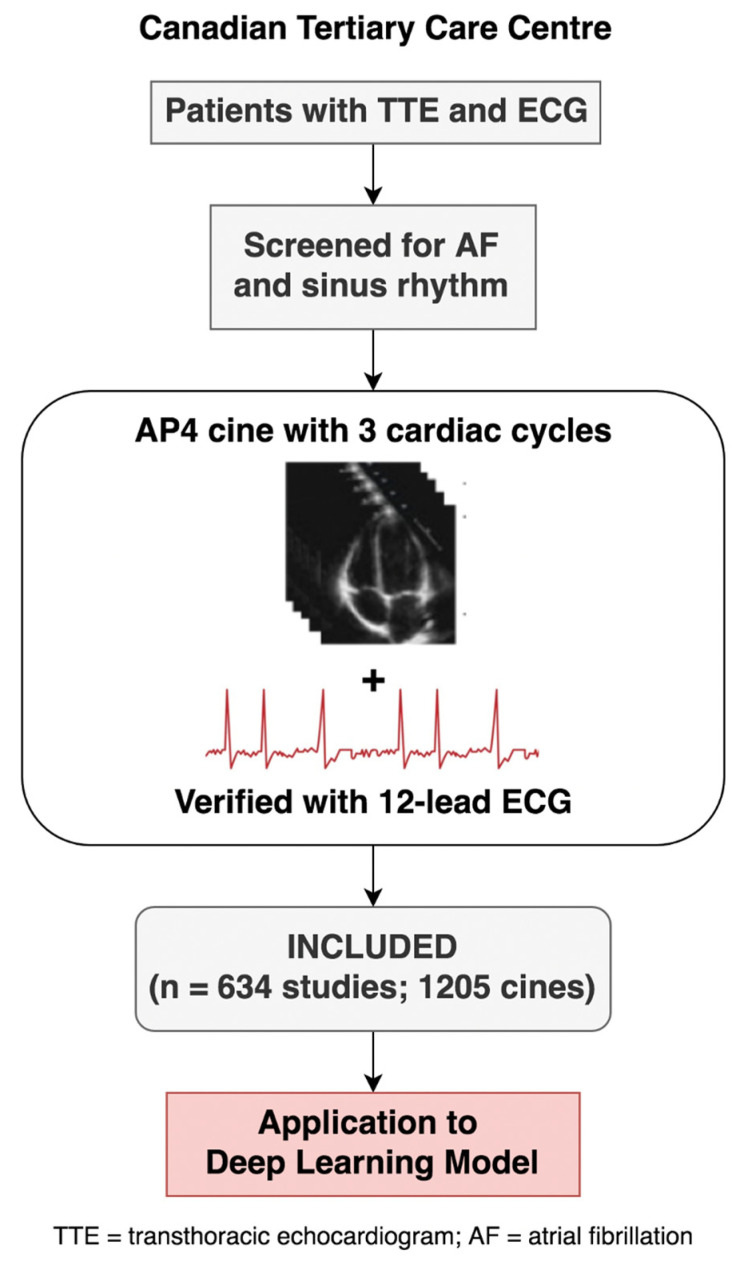
Study flow.

A total of 634 patient echocardiographic studies (1205 cines) met the inclusion criteria (Figure 2).

**Figure 3:**
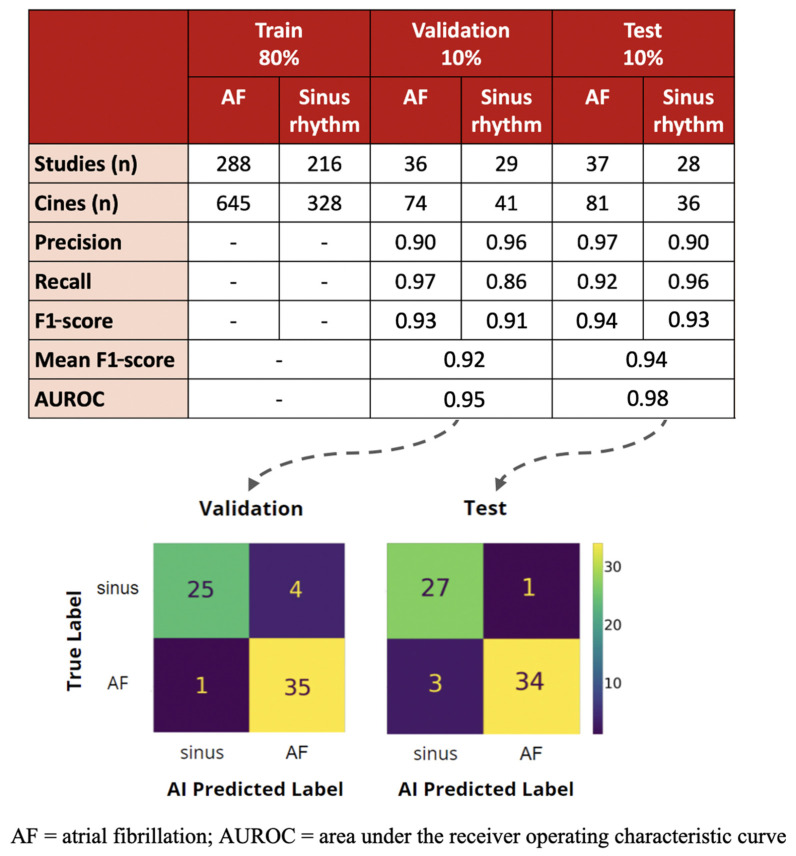
Evaluation of deep learning model on validation and test dataset.

The training dataset consisted of 288 studies (645 cines) for AF and 216 studies (328 cines) for sinus rhythm. The studies with AF had higher heart rates than those with sinus rhythm (84 ± 20 vs. 80 ± 19 bpm, *p* = 0.001), but there was no significant difference in the cine frame count (90 ± 34 vs. 90 ± 39 frames, *p* = 0.96) or duration (2.2 ± 0.5 vs. 2.2 ± 0.6 s, *p* = 0.69) of the three cardiac cycles used for the actual training process for AF and sinus rhythm, respectively. After training, the AI model achieved a mean F1-score of 0.92 and AUROC of 0.95 for rhythm detection on validation of 65 studies (115 cines), with individual F1-scores of 0.93 for AF and 0.91 for sinus rhythm (Figure 3).

### 3.2. Test Performance and Comparison to Echocardiography Cardiologist

When our trained AI model was subjected to 65 unseen studies (117 cines) of AF and sinus rhythm, it achieved F1-scores of 0.94 and 0.93 accordingly for an overall mean F1-score of 0.94 and AUROC of 0.98. Of the four cases where the AI model had predicted the incorrect rhythm, three were misdiagnosed as sinus rhythm and one was misdiagnosed as AF. However, further review of 12-lead ECGs for the case predicted as AF by the AI model reveal that the patient actually had paroxysmal AF as there were historical ECGs of the patient in AF, although they were in sinus rhythm at the time of the echocardiogram and thus had been labelled as such.

### 3.3. Qualitative Results with Occlusion-Based Importance Estimation

**Figure 4:**
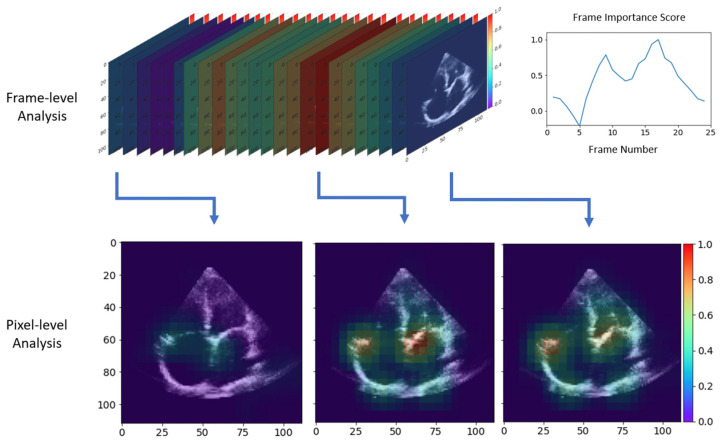
Heatmaps of potential echocardiographic features of atrial remodeling reflective of atrial fibrillation identified by the deep neural networks. Spectrum ranges from red being of high importance to violet being of least importance on analysis of cines in atrial fibrillation compared to sinus rhythm. **Top left** is a frame-by-frame analysis with a summary of frame importance on the top right. **Bottom** images are pixel analysis of select frames of high importance, showing attentiveness of the deep learning model to differences in atrial structure and function with a high focus on irregularities with the opening and closure of atrioventricular valves.

As shown in Figure 4 (and Supplemental Video S1), the frame-level analysis identified frames that captured the opening and closing of the atrioventricular valves as most important, as the exclusion of these frames from the input cine significantly reduced the accuracy of rhythm detection. This was further supported by the pixel-level analysis, which highlighted the regions closer to the tricuspid and mitral valves the most during periods of valve activity, with a particular emphasis on irregularities with their annular motion. Overall, these analyses demonstrated which anatomical regions and cardiac cycle phases the model focused on for predicting the rhythm of the given echo cine.

## 4. Discussion

To the best of our knowledge, this study is the first to confirm the accuracy of AF detection by AI using only echo images without accompanying rhythm strips or ECG. Our key findings are as follows: (1) the incorporation of an AI model in echocardiography is feasible for automated rhythm detection; (2) our trained AI model can identify AF with high, clinically acceptable accuracy and (3) differentiate from sinus rhythm on 2D AP4 cines without concurrent ECG or even Doppler information. Moreover, the AI model was shown to have consistent performance in these areas when compared to a level III-trained echocardiography cardiologists’ interpretation, the latter of which had access to the ECG rhythm strip and full study including Doppler information in addition to an external 12-lead ECG. The analysis of the cases where the AI model had predicted the incorrect rhythm suggests that some cases may be paroxysmal AF, whereby the AI model is detecting either anatomical or functional visual information consistent with AF that is not represented electrically on a standard 12-lead ECG or associated rhythm strip on echocardiography. These findings have immense implications as many thousands of echocardiography studies are performed daily on both cart-based and POCUS machines. The implementation of this AI AF detection feature will immediately accelerate our ability and accuracy for detection of AF worldwide.

We believe that this work is the first to integrate AI into echocardiography for the purpose of automated rhythm detection. We had previously used a semi-supervised deep learning-based framework called Echo-Rhythm Net to detect AF on 2D PLAX cines, with reported accuracy of 73–79% on the testing set [11]. Our present AI model using 2D AP4 cines appears to outperform our own prior model using Echo-Rhythm Net. We hypothesize two possible reasons for this observation: (1) Echo-Rhythm Net utilized a more complex convolutional neural network which could result in overfitting and make it more difficult for the AI model to perform accurately with unseen data; (2) the different echocardiographic views used for training, which was AP4 in this present study versus PLAX in Echo-Rhythm Net, may have been a factor as well, as changes in left atrial (LA) volume, function, and strain associated with AF can provide prognostic information [6,12,13]. A combination of apical and parasternal views is typically used in the routine assessment of the LA, and thus, both AI models have the opportunity to assess the LA as a marker of AF.

The use of AP4 view hypothetically provides more structural information relevant to AF which the AI model can learn from. Recent studies on right atrial and ventricular abnormalities have also been implicated in AF [14,15], and thus, it is possible that providing more cardiac chambers in view has allowed it to learn to detect AF with higher accuracy. Heatmaps derived from frame and pixel analysis of cines in AF compared to sinus rhythm indicate that the AI model is perceptive of changes in the atrial structure and function, with a particular focus on irregularities with the opening and closure of both tricuspid and mitral valves and their annular motion (Figure 4) (Supplemental Video S1). While these latter features appear to be deemed most important, closer examination reveals there is at least an intermediate level of importance along both atrial walls which infers assessment of atrial size. Altogether, this suggests that the AI model is potentially using echocardiographic features of atrial remodeling and function to phenotype AF. It is important to note that additional Doppler information was not provided to the AI model, which would have also allowed for the assessment of mitral inflow patterns in AF that are likely better assessed on AP4 view [6]. With that said, we acknowledge that PLAX is sometimes the easier view to obtain when acquiring imaging in practice due to patient positioning, consistent anatomical landmarks, and technical ease of holding the ultrasound probe in the appropriate position [16]. Our ongoing efforts to continue to increase the magnitude of our datasets to train our base AI model on PLAX cines, as well as combined AP4 and PLAX cines, will further enhance AF detection flexibility in the future.

At the level of dedicated echocardiography laboratories, AI may improve the assessment of AF with additional prognosticative ability and stroke risk stratification. Contemporary practice heavily relies on rhythm-based detection for diagnosis of AF followed by calculation of clinical scores such as CHADS2 or CHA2DS2-VASc. Yet, it is known that echocardiographic features such as large atrial size is a predictor of stroke independent of AF [17]. In fact, there has been an increase in the amount of research in the literature over the years suggesting that atrial myopathy is an independent predictor for stroke. The Cardiovascular Health Study was a large longitudinal study that investigated risk factors for cardiovascular disease and stroke in adults aged 65 years and older. The study found that markers for atrial myopathy including wave terminal force in lead V1 and N-terminal pro-B-type natriuretic peptide were associated with increased risk for thrombus formation and embolic stroke regardless of the presence of AF [18]. Subsequent studies on the use of strain imaging echocardiography have proposed that a positive global left atrial strain has prognostic value in assessing the severity of atrial myopathy and may further risk stratify patients with stroke [19]. Therefore, the echocardiographic severity of atrial remodeling potentially provides more information about AF burden and stroke risk than conventional ECG or Holter, which can be less sensitive for early or paroxysmal disease. With the aid of AI algorithms, this may allow echocardiography to detect AF faster and become a reliable adjunct to the current clinical scores for stroke risk stratification, allowing for more timely consideration of anticoagulation.

### Limitations

This study has important limitations. Our AI model performed exceptionally in the setting of our study, but its diagnostic accuracy may be affected by variable image quality in clinical practice especially when applied to POCUS. Differences in the specific ultrasound machine and operator experience will inevitably contribute to variable image acquisition. However, these differences can potentially be addressed with AI as well through recent advances in image analysis and optimization [8,20]. We acknowledge that the validation and testing datasets were relatively modest in size for the assessment of diagnostic accuracy. This was largely due to the 80:10:10 training–validation–test split ratio we chose for deep learning, where most studies were used for training purposes, and the overall echocardiographic studies available with three or more cardiac cycles for both AF and sinus rhythm to avoid cycle length being a confounding factor. Furthermore, our AI model utilizes a two-class classification layer which means it currently only identifies AF or sinus rhythm and has yet to be tested against other specific rhythms from a technical perspective. This is clinically relevant when attempting to differentiate AF from similar rhythms such as atrial flutter or irregularly patterns like sinus rhythm with premature atrial complexes or sinus arrhythmia. Given the early stages of developing this novel approach, our primary intent was for the AI model to first attain high accuracy in distinguishing AF from sinus rhythm and then build its library of rhythms successively. With that said, the AI model can be modified in a future sub-analysis to provide an uncertainty score when it is given a study that is outside of the distribution of AF or sinus rhythm. A high uncertainty score would indicate the AI model has determined a study is neither AF nor sinus rhythm. Finally, we recognize that the current AI model and the results attained are preliminary in terms of its applications to the real word. Nonetheless, we consider our work a successful proof-of-concept that supports continued effort in this field of AI in medicine. Future directions would be to prospectively validate our AI model with more datasets internally and externally outside our centre and to include POCUS imaging.

## 5. Conclusions

In summary, we have demonstrated that AI can automate rhythm detection on echocardiography without conventional ECG information. Our AI model can differentiate AF from sinus rhythm on 2D AP4 cines with a high diagnostic accuracy that appears to be comparable to that of an echocardiography cardiologist’s assessment of the ECG rhythm strip as the gold standard. This has major implications in POCUS where rhythm strips are unavailable and could help augment the clinical decisions of less experienced operators on diagnosis at bedside, leading to timely consideration of anticoagulation. Moreover, our AI model is potentially phenotyping AF based on atrial remodeling on echocardiography that is not reflected at the level of rhythm detection by ECGs, which may lead to earlier detection of AF and individualized stroke risk stratification for patients. Further prospective datasets and testing are required to validate this AI model to transition from proof-of-concept to real world applications.

## Data Availability

The datasets used to support the findings of this study are available from the corresponding author upon reasonable request.

## Supplementary Materials

The following supporting information can be downloaded at: https://www.mdpi.com/article/10.3390/diseases12020035/s1, Supplemental Video S1: Video of heatmap from Figure 4 of a cine in atrial fibrillation.
